# Preoperative neutrophil-to-lymphocyte ratio and tumor-related factors to predict lymph node metastasis in nonfunctioning pancreatic neuroendocrine tumors

**DOI:** 10.1038/s41598-017-17885-y

**Published:** 2017-12-13

**Authors:** Bo Zhou, Junfang Deng, Lifeng Chen, Shusen Zheng

**Affiliations:** 1 Department of Hepatobiliary Pancreatic Surgery, The First affiliated Hospital, School of Medicine, Zhejiang University, Key Lab of organ transplantation of Zhejiang Province, Key Lab of Combined Multi-organ Transplantation, Ministry of Public Health, Hangzhou, China; 2 Medical Engineering Department, The First affiliated Hospital, School of Medicine, Zhejiang University, Hangzhou, China

## Abstract

The lymph node (LN) status is very important for the survival in pancreatic neuroendocrine tumors (PNETs). Therefore, the investigation of factors related to LN metastases has a great clinical significance. The aim of this study was to evaluate the predictive value of the preoperative neutrophil-to-lymphocyte ratio (NLR), platelet-to-lymphocyte ratio (PLR) and possible clinical parameters on the LN metastases in nonfunctional PNETs (NF-PNETs). A retrospective review of 101 NF-PNET patients following curative resection and lymphadenectomy was conducted. The associations between clinicopathological factors and LN metastases and prognosis were determined. Twenty-seven (26.7%) patients had LN metastases. LN metastases was independently associated with disease-free survival (P = 0.009). Ideal cutoff values for predicting LN metastases were 1.80 for NLR, 168.25 for PLR and 2.5 cm for tumor size according to the receiver operating characteristic curve. On multivariable analysis, NLR (P = 0.017), symptomatic diagnosis (P = 0.028) and tumor size (P = 0.020) were associated with LN metastases. These results indicate that preoperative NLR ≥ 1.80, tumor size ≥2.5 cm and symptomatic diagnosis are independently associated with LN metastases for patients undergoing resection of NF-PNETs. It is anticipated that these findings are useful for further planning of lymphadenectomy before surgery.

## Introduction

Pancreatic neuroendocrine tumors (PNETs) are a heterogeneous group of neoplasms, accounting for approximately 1–2% of all pancreatic neoplasms and 7.0% of all neuroendocrine tumors^1^. The annual incidence of PNETs in the United States is estimated to range between 2 and 5 cases per one million individuals but appears to be rising, due to the application of imaging and endoscopic ultrasound^2^. PNETs can be classified as either functional or nonfunctional, while nonfunctional PNETs (NF-PNETs) account for 60% to 90% of all PNETs. Unlike functional PNETs with the typical clinical manifestations of hormone overproduction, NF-PNETs often had grown to an advanced stage with a large mass, local invasion and distant metastasis, because of the nonspecific symptoms in the early days, such as abdominal pain and distension, nausea and vomiting, abdominal mass, and others^3^. Complete surgical resection of a NF-PNET has been suggested to be the only potentially curative treatment for the disease, similar to pancreatic adenocarcinoma. The 5-year survival rate is about 40 to 60% with a median survival of 38 to 104 months^4,5^.

The current American Joint Committee on Cancer Staging (AJCC) and European Neuroendocrine Tumor Society (ENETS) considers tumor size, lymph node (LN) metastasis, and presence of distant metastasis in its staging criteria^6–8^. In addition, more and more evidence demonstrated that LN metastasis was an independent prognostic factor for PNETs^9,10^. Therefore, the investigation of factors related to LN metastases has a great clinical significance. However, preoperative factors predictive of LN metastases are not well defined in NF-PNETs. In recent years, markers of systemic inflammation, such as the neutrophil-to-lymphocyte ratio (NLR) and platelet-to-lymphocyte ratio (PLR), have been identified as prognostic factors. An elevated NLR and PLR have been shown to be correlated with advanced stages and poor prognosis in a variety of human tumors^11–13^. Tao L *et al*. reported that preoperative NLR, CA125 and CA19-9 are useful biomarkers for the prediction of LN metastasis in pancreatic ductal adenocarcinoma^14^. Whether preoperative NLR and PLR can predict the LN metastases of NF-PNETs remains unknown. Thus, we performed a retrospective analysis of predictor value of NLR and PLR and possible clinical parameters on the LN metastases of NF-PNETs before operation.

## Results

### Patient characteristics

A total of 101 patients with primary NF-PNET who underwent curative resection and lymphadenectomy were enrolled, including 53 males and 48 females. In this cohort, described in Table 1, the median age at the time of resection was 53 years, rang from 19 to 77 years. The most common presentation of the NF-PNETs was abdominal pain in 54 (53.5%) patients. Most tumors were located in the pancreatic body or tail (n = 57, 56.4%). Eighty-six patients (85.1%) underwent routinely formal resection (distal pancreatectomy or pancreaticoduodenectomy). The median size of NF-PNETs was 4.0 (range, 1.0 to 19.0) cm. Most tumors were of low or moderate grade (79.2%, grade 1 or 2), and 15 (14.9%) patients were classified as having distant metastasis at initial diagnosis.

**Table 1:** Clinical and pathological characteristics for patients with resected NF-PNET.

Variable	All patients (n = 101)
Age (yrs median[range])	53 (19–77)
Male gender (n [%])	53 (52.5%)
Presentation	
Abdominal pain	54 (53.5%)
Incidental finding	35 (34.6%)
Abdominal discomfort	6 (5.9%)
Jaundice	5 (5.0%)
Diarrhea	1 (1.0%)
Tumor size (cm median[range])	4.0 (1.0–19.0)
Location	
Head/uncinate/neck	44 (43.6%)
Body/Tail	57 (56.4%)
Surgical approaches	
DP	56 (55.4%)
PD	30 (29.7%)
Combined DP resection	5 (5.0%)
Combined PD resection	7 (6.9%)
Combined TP resection	3 (3.0%)
Grade	
G1	24 (23.8%)
G2	56 (55.4%)
G3	21 (20.8%)
Ki-67 index	
≤2%	28 (27.7%)
3 to 20%	52 (51.5%)
>20%	21 (20.8%)
Lymphovascular invasion (n [%])	34 (33.7%)
Positive lymph nodes (n [%])	27 (26.7%)
Distant metastasis at initial diagnosis (n [%])	15 (14.9%)

DP, distal pancreatectomy; PD, pancreaticoduodenectomy; Combined PD resection, pancreaticoduodenectomy with portal vein resection or hepatectomy or colectomy; Combined DP resection, distal pancreatectomy with portal vein resection or hepatectomy or colectomy; Combined TP resection, total pancreatectomy with portal vein resection or hepatectomy or colectomy.

### Clinicopathological features associated with LN metastases

**Figure 1:**
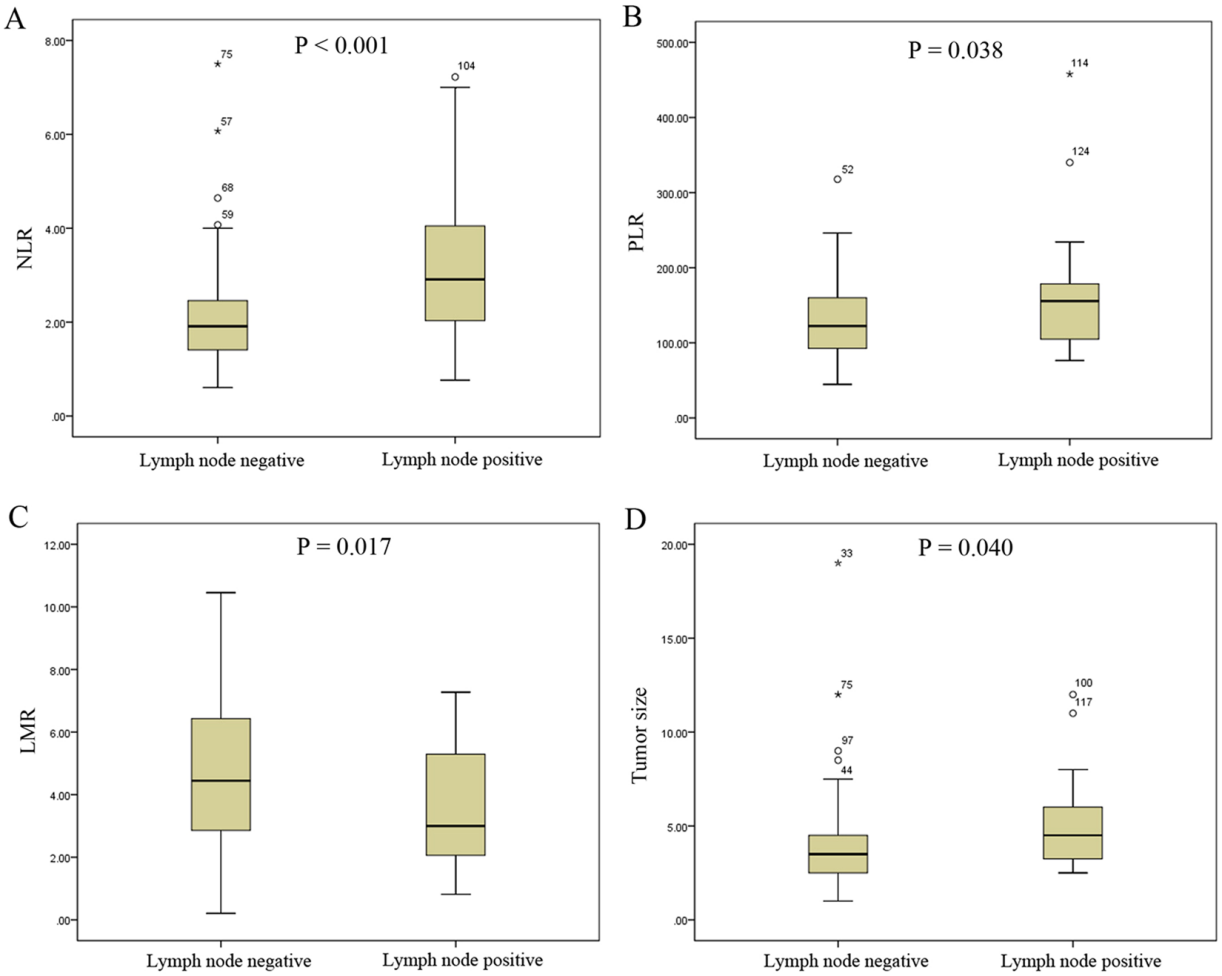
Distributions of NLR (**A**), PLR (**B**), LMR (**C**) and tumor size (**D**) between lymph node positive and lymph node negative.

Twenty-seven (26.7%) patients were discovered with LN metastases in the pathology. It demonstrated that both PLR and NLR were significantly higher in those patients with LN metastases, while lymphocyte-to-monocyte ratio (LMR) was significantly lower in those patients with LN metastases (all P < 0.05) (Fig. 1A,B and C). Whereas, it showed that the patients with LN metastases had larger tumor size (P = 0.040) (Fig. 1D).

**Figure 2:**
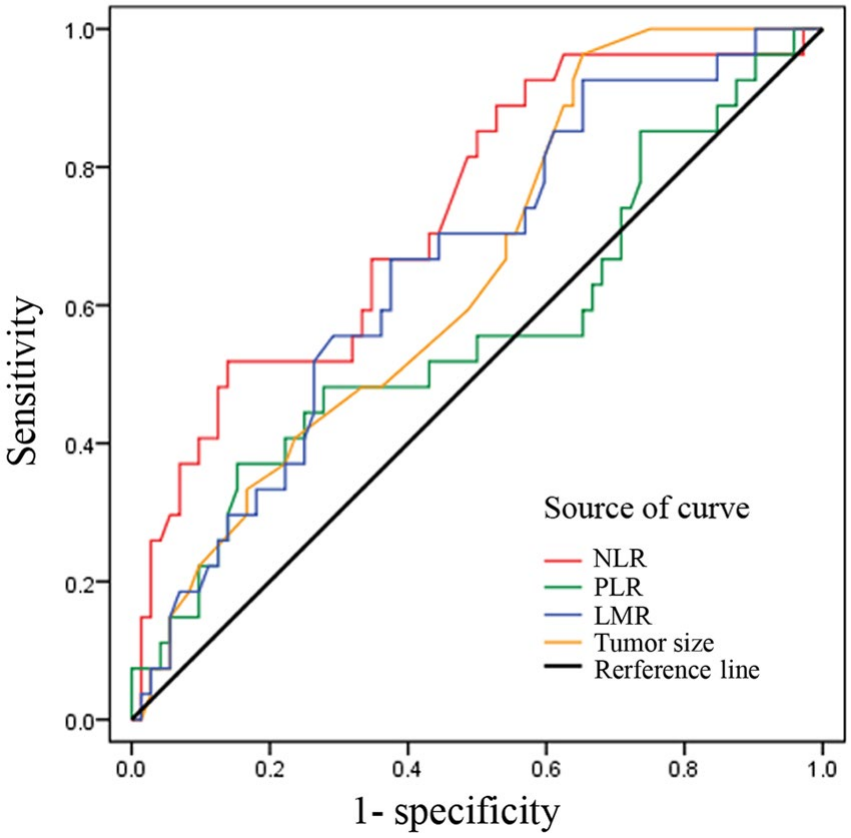
ROC curve for the NLR, PLR, LMR, and tumor size in resectable NF-PNETs.

ROC curve analysis showed that the AUCs of the NLR, PLR, LMR, and tumor size were 0.734, 0.565, 0.656, 0.647, respectively, and that the best cut-off values for the above parameters were 1.80, 168.25, 3.92, and 2.5, respectively, as these values were both the most sensitive and the most specific with respect to predicting LN metastases (Fig. 2). Preoperatively, the NLR was ≥1.80 in 62 (61.4%) patients, while the PLR was ≥168.25 in 22 (21.8%) patients. Additionally, the AUCs indicated that the ability of preoperative NLR values to differentiate LN metastases (sensitivity of 88.9% and specificity of 47.2%) was more powerful than others indicators.

As showed in Table 2, LN metastases was associated with NLR, PLR, LMR, alkaline phosphatase (AKT) level, tumor size, symptomatic diagnosis, Ki-67 index and vascular invasion (all P < 0.05). In the 62 patients with high NLR (≥1.80), the rate of LN metastases was 38.7%, while the rate of LN metastases for high PLR (≥168.25) was 45.5%. Of 83 patients with larger tumors (tumor size ≥2.5 cm), 27 (32.5%) patients had LN metastases, whereas all patients with small tumor had negative LN. However, gender, age, albumin, distant metastasis, tumor location, Eastern Cooperative Oncology Group performance status (ECOG-PS), and tumor markers including carbohydrate antigen 199 (CA199), carcinoembryonic antigen (CEA), alpha-fetoprotein (AFP), and carbohydrate antigen 125 (CA125) were not found to be associated with increased or decreased risk of LN metastases.

**Table 2:** Comparison of lymph node positive vs. lymph node negative patients.

Variables	Lymph node negative	Lymph node positive	Univariable analysis
	(n = 74)	(n = 27)	P
Age (years)			0.107
≤60	56	16	
>60	18	11	
Gender			0.94
Female	35	13	
Male	39	14	
Symptomatic diagnosis			**0.003**
No	32	3	
Yes	42	24	
Albumin (g/l)			0.566
<35	5	1	
≥35	69	26	
AKT (U/l)			**0.001**
<183	71	20	
≥183	3	7	
NLR			**<0.001**
<1.80	36	3	
≥1.80	38	24	
LMR			**0.01**
<3.92	28	18	
≥3.92	46	9	
PLR			**0.025**
<168.25	62	17	
≥168.25	12	10	
CA199			0.137
Normal	68	22	
Abnormal	6	5	
CA125			0.473
Normal	69	24	
Abnormal	5	3	
CEA			0.283
Normal	72	25	
Abnormal	2	2	
AFP			0.727
Normal	70	26	
Abnormal	4	1	
ECOG-PS			
0,1	68	23	0.318
≥2	6	4	
Tumor size (cm)			**0.005**
<2.5	18	0	
≥2.5	56	27	
Tumor location			0.055
Head/uncinate/neck	28	16	
Body/tail	46	11	
Distant metastasis			0.208
No	65	21	
Yes	9	6	
Ki-67 index			**0.001**
≤2%	27	1	
3 to 20%	37	15	
>20%	10	11	
Vascular invasion			**0.012**
No	67	19	
Yes	7	8	

AKT, alkaline phosphatase; NLR, neutrophil-to-lymphocyte ratio; PLR, platelet-to-lymphocyte ratio; LMR, lymphocyte-to-monocyte ratio; CA199, carbohydrate antigen 199; CEA, carcinoembryonic antigen; AFP, alpha-fetoprotein; CA125, carbohydrate antigen 125; ECOG-PS, Eastern Cooperative Oncology Group performance status. P-value < 0.05 marked in bold font shows statistical significant.

Multivariable logistic regression showed that the independent risk factors of LN metastases were NLR (HR = 6.218, 95% CI 1.390-27.821, P = 0.017), symptomatic diagnosis (HR = 4.979, 95% CI 1.185–20.922, P = 0.028) and tumor size (HR = 13.578, 95% CI 1.517–121.519, P = 0.020) (Table 3). All these factors, generally appealing to clinicians, can be reliably collected prior to surgery.

**Table 3:** Results of the preoperatively clinicopathological parameters for NF-PNET with lymph node metastasis by multivariate logistic analyses.

Variables	OR	95%CI	P
Symptomatic diagnosis			**0.028**
No	Reference		
Yes	4.979	1.185–20.922	
AKT (U/l)			0.336
<183	Reference		
≥183	2.720	0.355–20.841	
NLR			**0.017**
<1.80	Reference		
≥1.80	6.218	1.390–27.821	
LMR			0.982
<3.92	Reference		
≥3.92	0.983	0.223–4.335	
PLR			0.617
<168.25	Reference		
≥168.25	1.466	0.327–6.560	
Tumor size (cm)			**0.020**
<2.5	Reference		
≥2.5	13.578	1.517–121.519	
Ki-67 index			0.347
≤2%	Reference		
3 to 20%	5.089	0.518–50.027	
>20%	5.844	0.482–70.846	
Vascular invasion			0.244
No	Reference		
Yes	3.075	0.465–20.347	

NF-PNET, nonfunctional pancreatic neuroendocrine tumor; AKT, alkaline phosphatase; NLR, neutrophil-to-lymphocyte ratio; PLR, platelet-to-lymphocyte ratio; LMR, lymphocyte-to-monocyte ratio. P-value < 0.05 marked in bold font shows statistical significant.

### Comparison of the clinical variables in relationship to DFS after curative operation

**Figure 3:**
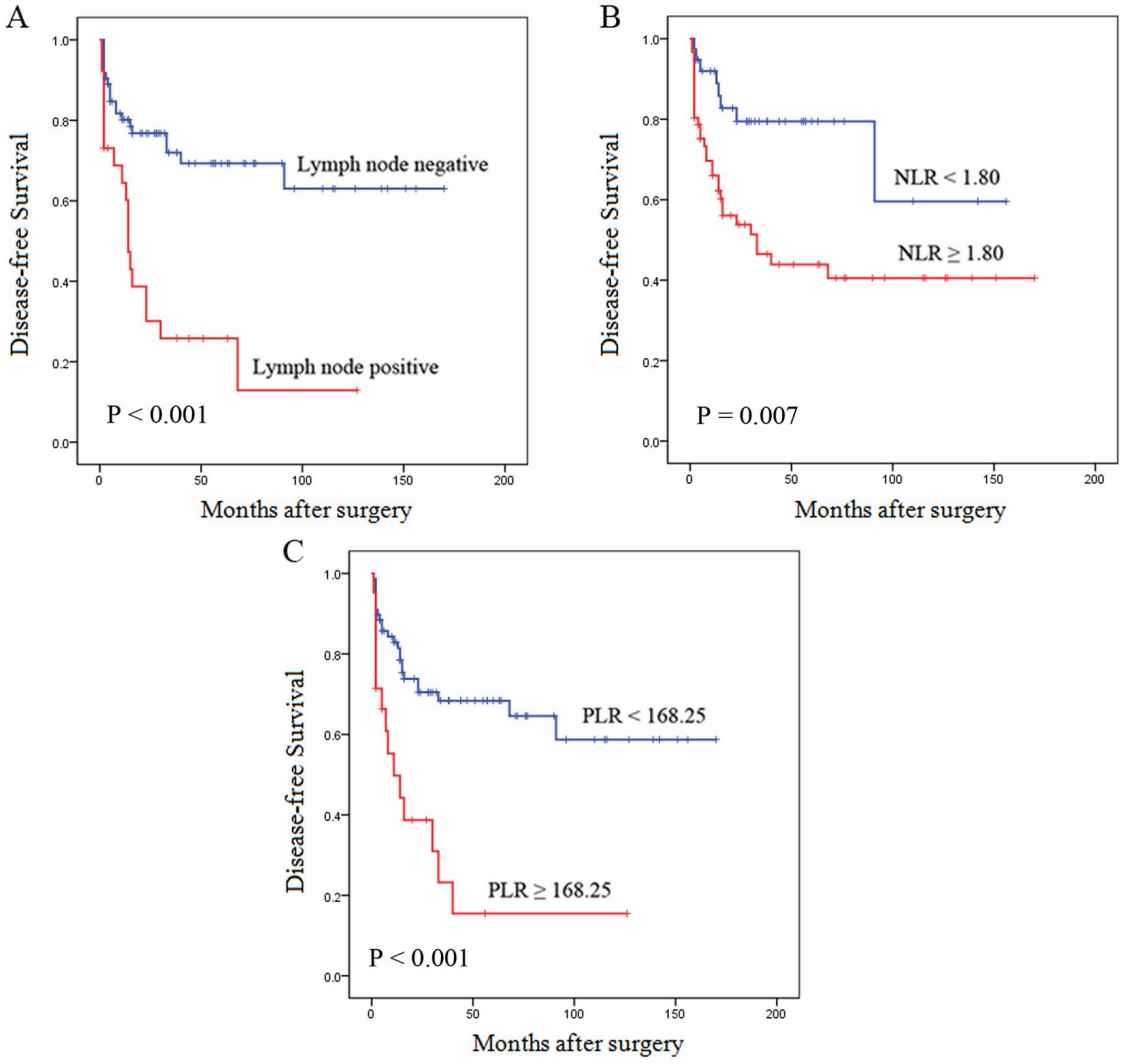
Disease-free survival for NF-PNETs stratified by lymph nodal status (**A**), NLR (**B**) and PLR (**C**). Lymph node positive, high NLR and high PLR are associated with poor survival (all P < 0.05).

Median duration of postoperative follow-up was 33 (range, 2 to 168) months. Thirty-nine patients (38.6%) had tumor recurrence during the follow-up. The results of the univariate disease-free survival (DFS) analysis for each of the clinicopathologic variables were shown in Table 4. NF-PNETs with LN metastases were about 4.6 times more likely to have tumor recurrence than patients without LN metastases (95% CI 2.467–8.627, P < 0.001) (Fig. 3A). Patients with a high NLR or PLR had shorter DFS than patients with a low NLR or PLR (Fig. 3B and C). Additionally, gender, AKT, Ki-67 index and vascular invasion were also prognostic factors for DFS (P < 0.05 for all). However, symptomatic diagnosis, LMR, tumor size, tumor location, albumin, age, CA199, CEA, AFP, CA125 and ECOG-PS were not significant predictors of DFS. Moreover, in the multivariate analysis, LN metastases (HR = 2.561, 95% 1.270–5.162, P = 0.009), PLR (HR = 2.310, 95% CI 1.134–4.708, P = 0.021), and Ki-67 index (HR = 9.088, 95% CI 2.377–34.755, P = 0.001) remained significantly associated with DFS.

**Table 4:** Variables associated with DFS according to the Cox proportional hazards regression model.

Variables	Univariable analysis		P	Multivariable analysis		P
	HR	95%CI		HR	95%CI	
Age (years)			0.144			
≤60	Reference					
>60	1.649	0.843–3.225				
Gender			**0.028**			0.066
Female	Reference			Reference		
Male	2.113	1.084–4.119		NA	NA	
Symptomatic diagnosis			0.059			
No	Reference					
Yes	2.050	0.973–4.321				
Albumin (g/l)			0.668			
<35	Reference					
≥35	1.366	0.328–5.685				
AKT (U/l)			**0.005**			0.803
<183	Reference			Reference		
≥183	3.063	1.398–6.711		NA	NA	
NLR			**0.007**			0.134
<1.80	Reference			Reference		
≥1.80	2.899	1.330–6.318		NA	NA	
LMR			0.094			
<3.92	Reference					
≥3.92	0.579	0.305–1.098				
PLR			** < 0.001**			**0.021**
<168.25	Reference			Reference		
≥168.25	3.359	1.748–6.455		2.310	1.134–4.708	
CA199			0.173			
Normal	Reference					
Abnormal	1.546	0.826–2.896				
CA125			0.675			
Normal	Reference					
Abnormal	1.205	0.505–2.875				
CEA			0.437			
Normal	Reference					
Abnormal	1.411	0.592–3.364				
AFP			0.767			
Normal	Reference					
Abnormal	1.153	0.45–2.951				
ECOG			0.74			
0,1	Reference					
≥2	1.191	0.424–3.364				
Tumor size (cm)			0.350			
<2.5	Reference					
≥ 2.5	1.482	0.650–3.381				
Tumor location			0.175			
Head/uncinate/neck	Reference					
Body/tail	0.794	0.570–1.108				
Ki-67 index			**<0.001**			**0.001**
≤2%	Reference			Reference		
3 to 20%	3.300	0.983–11.072		2.601	0.755–8.955	
>20%	12.681	3.627–44.336		9.088	2.377–34.755	
Vascular invasion			**0.002**			0.416
No	Reference			Reference		
Yes	3.224	1.541–6.741		NA	NA	
LN metastases			**<0.001**			**0.009**
No	Reference			Reference		
Yes	4.613	2.467–8.627		2.561	1.270–5.162	

DFS, disease-free survival; AKT, alkaline phosphatase; NLR, neutrophil-to-lymphocyte ratio; PLR, platelet-to-lymphocyte ratio; LMR, lymphocyte-to-monocyte ratio; CA199, carbohydrate antigen 199; CEA, carcinoembryonic antigen; AFP, alpha-fetoprotein; CA125, carbohydrate antigen 125; ECOG-PS, Eastern Cooperative Oncology Group performance status; LN, Lymph node. P-value < 0.05 marked in bold font shows statistical significant.

## Discussion

In the current study, we showed that 26.7% (27/101) NF-PNETs patients had LN metastases when diagnosed. Furthermore, LN metastases was associated with decreased DFS, which was similar to previous studies^9,10^. Interestingly, our study also showed that preoperative NLR ≥ 1.80, tumor size ≥ 2.5 cm and symptomatic diagnosis were independently associated with LN metastases for patients undergoing resection of NF-PNETs.

NLR, which can comprehensively reflect inflammatory and immune status in patients with cancer, has been a reliable marker for predicting the survival of patients with different types of tumor, such as PNET^15,16^, lung cancer^17^, colorectal cancer^18^, hepatocellular carcinoma^12^ and breast cancer^13^. Interesting, Lee *et al*. investigated the clinical impact of NLR as a prognostic factor in malignant pleural effusion and a new scoring system that use NLRs in the serum and malignant pleural effusion (smNLR score) in lung cancer patients. As a result, ECOG-PS (p < 0.001), histology (p = 0.001), and smNLR score (p < 0.012) were independent predictors of overall survival^17^. Regarding NETs, the report by Salman T *et al*. revealed that an elevated NLR and PLR were associated with a high tumor grade and an advanced tumor stage for NET. The study also verified that NLR and PLR were simple laboratory parameters that could be used to identify NETs with worse outcomes^19^. Recently, a retrospective analysis of 165 PNETs from Luo G *et al*. revealed that NLR was an independent predictor of overall survival for patients with PNETs. However, NLR > 2.4 was not significantly associated with positive lymph status (P = 0.067)^15^. In a retrospective study of 95 patients (21 functional PNETs and 74 NF-PNETs) undergoing resection for PNET, Tong Z *et al*. demonstrated that 15.8% (15/95) patients were histologically confirmed to have LN metastasis. The preoperative NLR was a potential independent predictor for LN metastasis and recurrence-free survival. The nomogram, including NLR, T stage, and grade, achieved a more optional performance in predicting LN metastasis, especially during the initial diagnosis for resectable PNET^16^. Both of the articles included functional PNETs or NF-PNETs, radical resection or non-radical resection. Since various studies have reported that function status and resection status are the independent predictors in patients with PNET, our study included 101 patients with NF-PNET who underwent curative resection to exclude these factors. Compared to functional PNET, NF-PNETs show a worse outcome in part due to the delay of diagnosis and higher malignant potential. Moreover, previous reports have demonstrated that only 30–40% of patients with NF-PNETs present with LN metastases at diagnosis, suggesting that most patients could be spared a lypmphadenectomy. In the present study, 26.7% (27/101) NF-PNETs patients had LN metastases when diagnosed. Addtionally, NLR ≥ 1.80 was associated with presence of LN positivity in 38.7% of patients, yet for NLR < 1.80, the rate of LN positivity was 7.7%. Further study revealed that NLR was an independent prognostic factor associated with LN metastases in patients with NF-PNET, which was similar with the results by Tong Z^16^. However, Arima *et al*. reported that NLR ≥ 2.4 predicted postoperative liver, but not lymph node, metastasis in PNET^20^. Heterogeneity across Arima and our studies may be attributed to several factors, such as differences in NLR cutoff values, differences in inclusion and exclusion criteria and differences in period of LN metastasis (LN metastasis during the surgery in our study and postoperative LN metastasis during the follow-up in Arima’s study). The best cut-off value for the NLR was based on the prediction of LN metastases in our study, while that in the research of Arima was based on the prediction of recurrence.

Increasing amounts of evidence have confirmed that tumor development is associated with inflammation and immunity. Inflammatory cells including leukocytes and lymphocytes play an important role in controlling proliferation, survival, and migration of tumor cells through apoptosis and angiogenesis pathways^21–23^. In addition, neutrophils, major part of WBCs, have a crucial role in tumor metastasis^24,25^. The study of Zhang J indicates that the abundance of circulating tumor-associated neutrophils in advanced cancer patients contributes to the tumor metastasis by inhibiting the activation of the peripheral leukocytes^26^. Other studies have indicated that tumor-associated neutrophils promote tumor proliferation, facilitate metastasis by releasing pro-angiogenic mediators (VEGF) and lead to more aggressive tumors^27^. It has also been pointed out that through interaction with neutrophils, tumor cells could be brought to the endothelium, which is an essential step in LN metastases. Wculek and Malanchi identify neutrophils as the main component and driver of metastatic establishment within the (pre-)metastatic lung microenvironment in mouse breast cancer models^28^. Furthermore, neutrophils can promote the adhesion of tumor cells to the lymphatic endothelium, which would bind to an endothelial cell if the endothelial is also sufficiently activated^29,30^. Therefore, neutrophils might be an important driver in LN metastasis.

Tumor size and symptomatic diagnosis, reliably available to a surgeon preoperatively, are also identified as predictors of LN metastases. A large number of researches have explored and reported on the LN positivity rates or progression rates at distinct size intervals: <1 cm 15%, <1.5 cm 13%, <2 cm 8–12%, <2.5 cm 8%, <3 cm 37%^9,31–34^. Tsutsumi K *et al*. reported increased prevalence of LN metastases in gastrinoma patients and non gastrinoma patients with tumor size ≥1.5 cm ^32^. They also found that 2 (8%) patients with gastrinoma out of 26 patients with tumor <1.5 cm had lymph nodal metastases^32^. Postlewait LM *et al*. also reported that tumor size ≥2 cm (HR = 6.52; 95% CI: 1.75–24.30; P = 0.005), male gender (OR = 3.16; 95% CI: 1.18–8.46; P = 0.02) and head/uncinate location (HR = 5.37; 95% CI: 2.07–13.96; P = 0.001) were associated with nodal-positivity. In addition, ROC analysis revealed that tumor size ≥ 2 cm was associated with nodal-involvement (AUC: 0.689; Sensitivity: 90%; Specificity: 53%)^35^. In contrast, Joyce Wong *et al*. reported that tumor size did not predict LN metastases. Furthermore, LN metastases did not impact OS or DFS, while tumor differentiation appears to be more important in determining prognosis^36^. In the current study, tumor size of ≥2.5 cm was associated with presence of LN positivity in 32.5% of patients, yet for tumors <2.5 cm, all the tumors had negative LN. In addition, we also found that patients who were symptomatic at diagnosis were more likely to have LN metastases, compared to incidentally diagnosed NF-PNETs (P = 0.003). However, there were still 3 cases out of 35 incidentally diagnosed patients (8.6%) had LN metastases. Our data suggested that tumor size was more useful to predict LN metastases, while NLR and symptomatic diagnosis could not reliably predict LN metastases.

Our study had several limitations that must be considered. First, given its retrospective design, the current study was subject to possible selection bias, as well as diagnostic bias. Second, the NLR and PLR, a marker of systemic inflammation, may be affected by many conditions, including chemotherapy toxicity, chronic inflammatory diseases, granulocyte colony-stimulating factor administration, pathogenic inflammation and other diseases. Therefore, these conditions must be accounted for in clinical practice. Finally, the present study was conducted at a single institution. The performance of multicentre studies of the markers used herein would strengthen our conclusions.

In conclusion, this study highlights that NLR ≥ 1.80, tumor size ≥ 2.5 cm and symptomatic diagnosis are independently associated with LN metastases for patients undergoing resection of NF-PNETs. It is anticipated that these findings are useful for further planning of lymphadenectomy before surgery.

## Material and Methods

### Study population

Patients who underwent surgical resection and lymphadenectomy for NF-PNETs from November 2003 to August 2016 at the First Affiliated Hospital, Zhejiang University School of Medicine, were retrospectively reviewed. The diagnosis of NF-PNET was made based on standard histologic criteria. The TNM stage of each PNET was determined based on the American Joint Committee on Cancer TNM Classification, while the grade of each PNET was determined according to the 2010 WHO classification of NETs of the GEP system. Patients who showed clinical evidence of infection or evidence of hyperpyrexia at the time of diagnosis (including positive bacterial culture, cholangitis) were excluded from the study (n = 8), as were patients who received preoperative radiochemotherapy (n = 2) and who had a history of cancer of any type (n = 3). We included only those patients who had survived for at least 60 days after surgery in the study to exclude perioperative mortality-related bias. Finally, 101 patients undergoing curative resection and lymphadenectomy were enrolled.

The radiological examination before operation included ultrasonography, abdominal computed tomography and magnetic resonance imaging. Since 2012, the endoscopic ultrasonography or endoscopic ultrasonography guided fine needle aspiration biopsy has been performed in some patients, whose diagnosis was indistinct. Radical resection was considered the first-choice treatment for patients with PNET. For the nonmetastatic PNET patients undergoing radical resection, no postoperative somatostatin analogue therapy, targeted therapy or systematic chemotherapy was carried out. In patients presenting with metastatic PNET, multiple treatment modalities were used after operation, including somatostatin analogue therapy and systematic chemotherapy. Laboratory tests including blood routine, tumor markers and liver function were routinely performed within 7 days before the surgical resection. The NLR was calculated by dividing the absolute neutrophil count by the absolute lymphocyte count. The PLR was calculated by dividing the absolute platelet count by the absolute lymphocyte count, while LMR was calculated by dividing the absolute lymphocyte count by the absolute monocyte count on preoperative routine blood tests. Meanwhile, we defined normal values of CA199, CEA, AFP, and CA125 as 0–37 U/ml, 0–5 ng/ml, 0–20 ng/ml, and 0–35 U/ml, respectively. ECOG-PS is an ordinal scale with scores from 0 to 5: 0, normal activity; 1, symptomatic but ambulatory; 2, symptomatic-confined to bed/chair < 50% of waking hours; 3, symptomatic-confined to bed/chair > 50% of waking hours; 4, 100% bedridden; and 5, dead. The study was approved by the Ethics Committee of the First Affiliated Hospital of Zhejiang University School of Medicine and conducted in accordance with the Declaration of Helsinki. Written informed consent was obtained from all participants before the commencement of the study. All methods and research activities were performed in accordance with the guidelines and regulations.

### Follow-up

Patient follow-up was performed by reviewing hospital records or contacting patient family members. Overall survival (OS) was defined as the time span extending from the date of initial diagnosis until the date of death from any cause or the date of last known contact. Disease-free survival (DFS) was calculated from the day of surgery until the time of recurrence. Our department follows up with patients every 6 months for the first 5 years after surgery and then yearly thereafter. The following postoperative follow-up data were collected for each patient: clinical symptoms and signs, laboratory test results and radiological examination results. Once recurrence was confirmed, patients were treated by repeat tumor resection, radiofrequency ablation (RFA), transarterial chemoembolization (TACE), systematic chemotherapy and somatostatin analogue therapy, according to the sizes, numbers and locations of their recurrent tumors. And no patients received the targeted therapy including everolimus or sunitinib.

### Statistical analysis

All statistical analyses were performed using SPSS 16.0 software (SPSS, Chicago, IL, USA) for Windows. Differences in the NLR and PLR and other clinicopathologic features between positive LN and negative LN were evaluated by t tests in the case of normally distributed variables or by the Mann-Whitney U test in the case of abnormally distributed variables. Area under the curve (AUC) values obtained from receiver operating characteristic (ROC) curve analysis were used to compare the predictive efficacies of NLR and PLR. The associations between the clinical and histopathological parameters with LN metastases were evaluated by both univariate analysis and multivariate logistic regression analysis. The Kaplan-Meier method and the log-rank test were used to calculate DFS. Prognostic analysis was performed using univariate and multivariate Cox regressions models. A P value < 0.05 was considered statistically significant. All data generated or analysed during this study are included in this published article.
